# The design of a history of pharmacy course in the bachelor of pharmacy curriculum in the UAE experience

**DOI:** 10.5116/ijme.56d5.aa8d

**Published:** 2016-03-12

**Authors:** Bazigha K. Rasool, Sabeena Salam

**Affiliations:** 1 Department of Pharmaceutics, Dubai Pharmacy College, Dubai, UAE; 2 General Education, Dubai Pharmacy College, Dubai, UAE

**Keywords:** history of pharmacy, bachelor of pharmacy, pharmacy curriculum, UAE experience, pharmacy course

## Introduction

The study of the history of pharmacy is deserving of consideration as a review of the past, so that we may understand the present, and thus be enabled to plan intelligently for the future.^1^ However, inclusion of this syllabus has suffered a crushing blow in the global context. For example, in the US, Buerki (1981) conducted a far-reaching survey and reported that almost 40% of pharmacy schools offered either required or elective course work in the history of pharmacy, 32% offered orientation courses that contained some historical component, and 29% offered no such course work. Notably, there was a significant decline in the number of pharmacy schools offering this course. Buerki's findings may have prompted the American Council on Pharmaceutical Education to adopt a strong policy statement on teaching the history and social studies of pharmacy later that same year.^2^ Even in the past, Edward Kremers remarked parenthetically in an 1892 address before the American Public Health Association (APHA) Section on Pharmaceutical Education and Legislation that, "the professional student should at least have a fair knowledge of this history of his profession”.^3^

### What is the problem?

In the UAE, little attention is given to historical aspects of pharmacy as a standalone course in the pharmacy curriculum.^4^^-^^7^ In other words, until recently, the course History of Pharmacy has not been introduced in BPharm or PharmD curricula offered by pharmacy colleges, including Dubai Pharmacy College (DPC) - the first pharmacy college in the region established in 1992. In recent times, the Commission for Academic Accreditation, Ministry for Higher Education and Scientific Research, UAE; specifies in the 2011 “Standards for Licensure and Accreditation”, section that the institutions must ensure that all undergraduate students complete the equivalent of one or more university level courses in the humanities, appropriate to the program offerings.^8^ In compliance, DPC designed a course titled “History of Pharmacy” in the domain of Humanities. This course is offered to the BPharm students with the purpose of acquiring knowledge and critical thinking skills to understand a variety of perspectives and diverse historical experiences resulting in enhancing aspects of competence in terms of: autonomy and responsibility, role in context, and self-development.^9^

### What we have done?

At an early stage among pharmacy students, a compulsory course entitled “History in Pharmacy” is contextualized and designed at Dubai Pharmacy College. The course is given to first year students from the academic session 2015-2016. Topics include,

   •   The stages of development in pharmacy profession in Europe, United States, Middle East and Far East regions,

   •   The significant role of the ancient world - Mesopotamian, Egyptian, Greek, and Roman - in the progress of making medicine,

   •   The outcomes of discovery of critical medicines that revolutionized patient and disease management and how this affected the practice in the profession,

   •   The global growth of professionalism in the field of community pharmacy, hospitals and pharmaceutical industry,

   •   Important factors and events that shaped the profession of pharmacy in Gulf Countries particularly in the UAE.

For the purpose of accreditation, the course got approved by the Commission for Academic Accreditation (CAA), Ministry for Higher Education and Scientific Research, UAE.

### What lessons were learned?

In our utilitarian and materialistic age, such a course enables pharmacist students to develop their knowledge about the early history of pharmacy and understand that pharmacy is a part of the larger context of socio-political, socio-economic, and socio-cultural development in which it resides.^8^ Needless to say, immense efforts are needed to reinforce the topics being discussed. Technical aids are very useful to create a more interesting and beneficial teaching environment. For instance, power-point slides, video demonstrations and materials on the internet enable students to visualize and experience something that is impractical to see or do in real life. It is planned that “history of pharmacy” is going to be offered as a team-taught course.  The collaborative team teaching approach allows students to observe high-level intellectual debate among colleagues and to increase the amount of feedback they receive from instructors.^10^

## Conclusions

Our observations convinced us that a specific course in the history of pharmacy, in pharmacy education, offered as a compulsory course in the domain of humanities is much more effective in developing a conscious appreciation of the importance and value of the historical developments of pharmacy profession. Most importantly, such a course has potential to ‘shape and/or transform’ the healthcare landscape for the benefit of humanity. In summary, the format of the course may be different, but learning the historical aspects of pharmacy is one of the keys to personal and professional development.

### Acknowledgements

The authors would like to express their sincere thanks to the Dean Prof. Saeed Ahmad Khan of Dubai Pharmacy College.

### Conflict of Interest

The authors declare that they have no conflict of interest.
